# Patient reported pain following tooth extraction with different autologous platelet concentrates. Systematic review

**DOI:** 10.1038/s41405-025-00348-2

**Published:** 2025-07-17

**Authors:** Haidar Hassan, Rawand Shado, Ines Novo Pereira, David Madruga

**Affiliations:** 1 Rey Juan Carlos University, Av. de Atenas, S/N, 28922 Alcorcón, Madrid, Spain; 2 Barts & The London School of Medicine & Dentistry, Queen Mary University, Centre for Cutaneous Research, Blizard Institute of Cell and Molecular Science, 4 Newark Street, Whitechapel, London, E1 2AT UK; 3 Barts & The London School of Medicine & Dentistry, Queen Mary University, Institute of Dentistry, Royal London Dental Hospital, Turner Street, London, E1 2AD UK; 4 Faculty of Dental Medicine, University of Porto (FMDUP) Rua Dr. Manuel Pereira da Silva, 4200-393 Porto, Portugal; 5 Egas Moniz Center for Interdisciplinary Research (CiiEM); Egas Moniz School of Health & Science, Campus Universitário, Quinta da Granja, 2829-511 Caparica, Almada Portugal

**Keywords:** Third molar removal, Dentoalveolar surgery

## Abstract

**Background:**

Autologous platelet concentrates (APCs) have played a significant role in regenerative dentistry, with clinical evidence suggesting its benefits over controls. Particularly, APCs could reduce postoperative pain following tooth extractions.

**Aim:**

To compare patient reported pain after tooth extractions using different autologous platelet concentrates (APCs) such as platelet-rich plasma (PRP) and platelet-rich fibrin (PRF).

**Method:**

A search on Pubmed, Scopus, Embase and Google Scholar databases was conducted to identify human studies using APC(s) in extraction sockets between January 2014 and June 2024. This review followed the PRISMA guidelines. The inclusion criteria involved comparative human studies ranging from evidence levels II to III (Oxford Centre for Evidence-Based Medicine Levels of Evidence). For assessing bias in the included studies, the Cochrane Risk of Bias tools were used. The Grading of Recommendations Assessment, Development and Evaluation (GRADE) approach was used to determine the quality of evidence available.

**Results:**

This review identified 8 studies; with 338 extraction sites in total and 1–15 days pain follow up. Four studies showed no statistically significant difference in postoperative pain reduction between PRP and PRF. One study observed no statistically significant difference between leukocyte-rich PRF (L-PRF) and titanium-prepared PRF (T-PRF). One study indicated that advanced platelet-rich fibrin (A-PRF) is superior to PRF in reducing postoperative pain on day 2 postoperatively. In addition, two studies reported that A-PRF is more effective than L-PRF on day 2. Moderate-to-high risk of bias was identified within 75% of the selected papers. GRADE score for evidence quality assessment was ‘Low’.

**Conclusion:**

A-PRF was favoured to reduce postoperative pain on day 2 among the investigated APCs, although the GRADE criteria rate the evidence as “Low”. Future trials should directly compare A-PRF with PRF and L-PRF using high-quality randomized controlled designs.

## Introduction

### Autologous platelet concentrates (APCs)

In recent years, autologous platelet concentrates (APCs) have played a significant role in regenerative dentistry, particularly in applications such as sinus floor elevation, peri-implantitis, medication-related osteonecrosis of the jaw (MRONJ), bone regeneration, and socket preservation [1, 2].

**Fig. 1:**
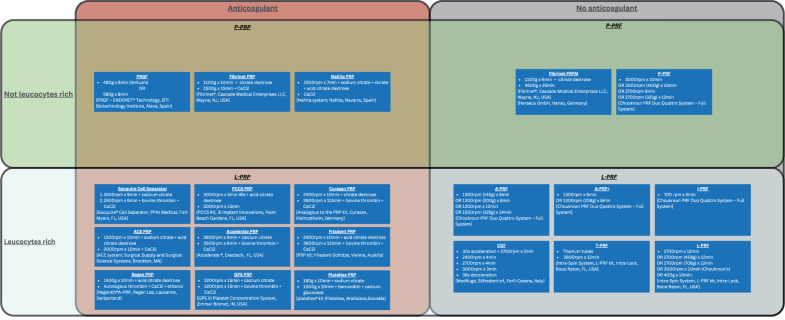
Classification and characteristics of autologous platelet concentrates. The boxes within each section provide specific details about the preparation protocols, including centrifugation speeds, durations, added substances and the centrifuge machine.

The first APC, platelet-rich plasma (PRP), was introduced in the field of haematology for transfusion purposes [3]. In 1990s, PRP was firstly used in oral and maxillofacial surgery [4]. It took approximately a decade for Choukroun to introduce the platelet-rich-fibrin (PRF) [5], leading to the development of various APC subcategories with and without anticoagulants, as well as formulations rich or poor in leucocytes [6]. These subcategories are further differentiated into unique formulations based on distinct preparation protocols [7, 8] (Fig. 1).

The literature supports that the main components of platelet concentrates for facilitating healing and repair processes are leucocytes and growth factors [9]. Upon activation, these growth factors, embedded within the fibrin matrix, have been shown to stimulate a mitogenic response in periosteal cells, promoting bone healing [10].

### Socket preservation

Following tooth extraction, the supporting bone undergoes resorption [11], with two-thirds of the surrounding bone affected within the first three months [12]. Adequate bone levels are essential for the survival and long-term stability of dental implants [13, 14]. To mitigate bone loss, socket preservation strategies with biomaterials such as bone grafts and membranes are employed [15]. Numerous approaches to socket preservation have been developed over time [16], including the use of APCs like PRP and PRF [17]. Systematic reviews have demonstrated that APCs are effective in reducing vertical bone resorption following tooth extraction [17–19].

### Pain following tooth extraction

Postoperative pain following tooth extraction is a common risk, persisting for up to seven days [20]. Key risk factors include oral hygiene, procedural difficulty, operator skill level, smoking status, age and the use of oral contraceptives [21]. A recent systematic review of 82 studies identified that the combination of ibuprofen and acetaminophen or naproxen, as well as oxycodone and acetaminophen, are the most effective oral analgesics for managing pain post-tooth extraction [22]. Nevertheless, oral analgesics may be insufficient for some patients [23], prompting the investigation of adjunctive interventions to further alleviate postoperative pain.

Various methods to mitigate postoperative pain have been studied, including photobiomodulation [24], submucosal injection of dexamethasone [25], and low-level laser therapy [26]. However, these interventions do not provide the additional benefits of enhanced healing and socket preservation observed with the use of APCs [27, 28].

### Rationale behind this review

Conventionally, oral analgesics are routinely used to reduce postoperative pain following tooth extraction. However, given that oral analgesics are not always sufficient and considering the added benefits of APCs, such as promoting healing and socket preservation, it is reasonable to consider APCs as adjunct interventions for postoperative pain management. Therefore, identifying the most effective APC for this application is warranted.

The aim of this evidence-based review was to evaluate the current evidence to identify the best APC for reducing postoperative pain following tooth extraction.

## Methods

The Preferred Reporting Items for Systematic reviews and Meta-Analyses (PRISMA) was followed for reporting this review (Supplement 1) [29]. The PICO framework was used to structure the reporting of eligibility criteria [30]:

(P) Population: Controlled clinical studies involving tooth extractions

(I) Intervention: Application of an APC in extraction sockets

(C) Comparison: Application of another APC in extraction sockets

(O) Outcomes: Patient self-reported pain

### Search strategy

PubMed, Scopus, Embase and Google Scholar were the main databases used for conducting the search for articles in this review. Table 1 presents the key search terms used for articles retrieval. The databases were searched between 1^st^ of January 2014 and June 24th, 2024. The search was confined to peer-reviewed journal articles indexed in the selected electronic databases; grey-literature sources were not explored.

**Table 1:** Search key words.

PubMed search	Scopus	Embase	Google Scholar search
(platelet*) AND (extract* OR surger* OR remov*) AND (socket* OR molar* OR teeth OR tooth OR incis* OR canin* OR premolar*)	TITLE-ABS-KEY ((platelet*) AND (extract* OR surger* OR remov*) AND (socket* OR molar* OR teeth OR tooth OR incis* OR canin* OR premolar*))	(platelet*) AND (extract* OR surger* OR remov*) AND (socket* OR molar* OR teeth OR tooth OR incis* OR canin* OR premolar*)	(platelet OR platelets) (extraction OR extracted OR extractions OR removed OR removal) (socket OR sockets OR molar OR molars OR teeth OR tooth OR incisor OR incisors OR canine OR canines OR premolar OR premolars)

A table showing the search strategy employed in PubMed, Scopus, Embase and Google Scholar to identify the articles for this review.

### Study selection

The eligibility criteria (Supplement 1b) ensured that the selected studies focused on comparing different APCs in reducing postoperative pain following tooth extraction.

Study selection was conducted by two independent reviewers (HH, RS) in the following stages: (1) Initial screening of potentially suitable titles and abstracts against the inclusion criteria to identify potentially relevant papers. (2) Screening of the full papers identified as possibly relevant in the initial screening. (3) Studies were excluded if not meeting the inclusion criteria. Following the screening of titles and abstracts, the studies included by both reviewers were compared. In case of a disagreement between reviewers, the decision about study eligibility was made by trying to reach a consensus between the two reviewers. In case of continued disagreement, a third reviewer (INP) judged study inclusion.

### Data collection

In relation to each investigated study, data collection was completed independently by two reviewers (HH, RS). Another author (INP) reviewed extracted data and resolved any discrepancies.

**(1) Study Publication Details**: Authorship, year of publication, and country of origin. (2) **Study Characteristics**: Demographic variables including extraction sites, sex and age. (3) **Study Methodology**: APCs employed, APC preparation protocols and investigated outcomes. The selected studies were categorised using the Oxford Centre for Evidence-Based Medicine Levels of Evidence (OCEBM) classification system [31]. (4) **Study Outcomes**: Patient reported pain.

### Data preparation

In instances where a specific data point was entirely absent, we systematically documented and presented this absence as “Not Reported” (NR) in our analysis. Regarding continuous variables, such as age, when distinct values were provided for each respective group and there were no statistically significant difference between the groups, we combined mean and standard deviation values into our table.

### Risk of bias

The Risk of Bias 2 (RoB2) assessment tool used to assess risk of bias for RCTs (Level II) [32], and the Risk of Bias tool of Non-randomised Studies - of Interventions (ROBINS-I) for cohort studies (III) [33]. The bias category of ‘Some concerns’ was labelled as ‘Moderate’ for the purpose of font size clarity in the generated tables.

For each study the overall bias was given based on the highest bias score for each decision category. For example, if the highest score of ‘high’ was estimated for one or more decision categories, then the overall bias was considered ‘high’.

### Data analysis

The Grading of Recommendations Assessment, Development, and Evaluation (GRADE) approach [34] was used to determine the quality of evidence available in the identified studies for this evidence-based review [34]. Each component of the GRADE approach was independently assessed by two reviewers (RS, HH). In cases of disagreement, another author (INP) facilitated a discussion to ensure consensus was reached. It was anticipated that the nature of patient-reported outcome measurement would lead to high heterogeneity in results and methodologies, hence, a quantitative meta-analysis would not be feasible. Therefore, a qualitative comparison network was employed alternatively.

## Results

### Studies included

**Fig. 2:**
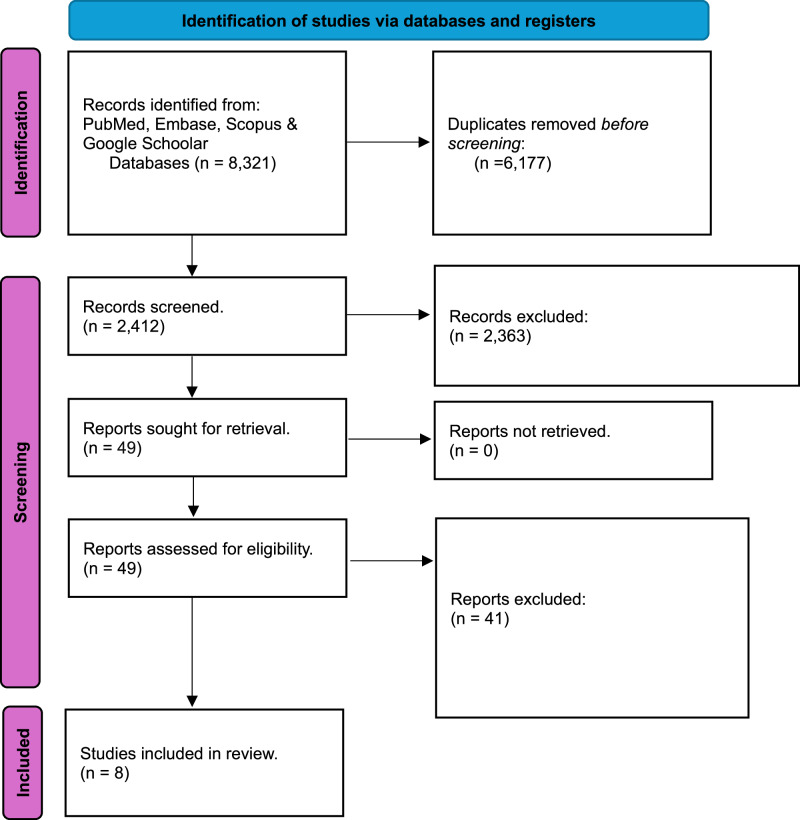
PRISMA flowchart. A flowchart demonstrating the identification, screening and the inclusion process of the included articles in this review.

Figure 2 shows the PRISMA flowchart representing study selection and inclusion [29]. The initial search resulted in 8321 papers for all databases combined. This was trimmed down to 2412 after duplicates were removed. Following the first-stage screening of titles and abstracts, 49 articles (considered potentially suitable by at least one reviewer) qualified for full-text screening. After full-text reading; 8 studies, with 338 extraction sites in total and 1–15 days pain follow up, met the inclusion criteria, and 41 papers were excluded.

### Study characteristics

Table 1 reports the studies and their characteristics, 4 RCTs and 4 cohort studies [35–42]. The table shows the authors, year of publication, country of publication, study type, level of evidence, risk of bias, APC preparation protocol, groups characteristics, outcomes measured and pain results. Studies were classified as level of evidence II for RCTs and level III for cohort studies (Table 2).

**Table 2:** Characteristics of the included studies.

Authors, year, country	Study type (level of evidence) - Risk of bias	Population Characteristics	Sites involved	Groups	Concentrates preparation	Outcomes Measured	Pain results	Favoured protocol	Funding
Yari et al., [42], Iran	RCT (II) - Moderate	Sites: 62 Males: 28 Females: 34 Age: 22.5 ± 3.2	Mandibular Third Molars	Sites: 15 Protocol: Control Sites: 15 Protocol: A-PRF Sites: 16 Protocol: A-PRF+ Sites: 16 Protocol: L-PRF	L-PRF: 2800 rpm x 12 min (TD 4, HereXi Instruments Inc.) A-PRF: 1500 rpm x 14 min (TD 4, HereXi Instruments Inc.) A-PRF + : 1300 rpm x 8 min (TD 4, HereXi Instruments Inc.)	soft tissue healing, pain, analgesic use, trismus, facial swelling, and alveolar osteitis.	VAS Scores at 1, 2 and 3 days: Control: 48 ± 11.86, 36.13 ± 7.72, 17.38 ± 7.72 L-PRF: 38.13 ± 5.43, 30.31 ± 3.04, 10.63 ± 4.42 A-PRF: 34.38 ± 5.25, 23.69 ± 7.72, 7.88 ± 4.29 A-PRF + : 34.69 ± 5.62, 25 ± 6.58, 9.13 ± 4.36	A-PRF and A-PRF +	None
Riaz et al., [41], India	Cohort Study (III) - High	Sites: 20 Males: 3 Females: 7 Age: 26.5 (18– 35)	Mandibular Third Molars	Sites: 5 Protocol: PRF Sites: 5 Protocol: A-PRF Sites: 10 Protocol: Control	A-PRF: 1500 rpm x 14 min (REMI C-852, Remielektrotechnik Ltd, Vasai, India) PRF: 3000 rpm x 10 min (REMI C-852, Remielektrotechnik Ltd, Vasai, India)	Pain, swelling, and mouth opening (VAS for pain, flexible tape for swelling, interincisal distance for mouth opening).	VAS Scores at 1, 3 and 7 days: PRF control: 2.6, 2.0, 1.6 PRF: 2.4, 1.4, 0.6 A-PRF control: 3.0, 2.4, 1.4 A-PRF: 2.6, 1.4, 0.4	A-PRF	None
Verma et al., [40], India	Cohort Study (III) - High	Sites: 40 Males: 13 Females: 7 Age: (21–48)	Mandibular Third Molars	Sites: 20 Protocol: PRF Sites: 20 Protocol: PRP	NR	Pain using 1-10 rating communicated by patient, trismus, and swelling	Pain scores mean of 15 days: PRF: 3.83 ± 0.456 PRP: 3.42 ± 0.536	none	NR
Ustaoğlu et al., [39], Turkey	RCT (II) - Low	Sites: 57 Males: 28 Females: 29 Age: 35.4 ± 5.6	Anterior and Premolar teeth	Sites: 19 Protocol: Control Sites: 19 Protocol: T-PRF Sites: 19 Protocol: L-PRF	L-PRF: 2700 rpm x 12 min (Intra-Spin System, L-PRF kit, Intra-Lock, Boca-Raton, FL, USA) T-PRF: 2800 rpm x 12 min (Intra-Spin System, L-PRF kit, Intra-Lock, Boca-Raton, FL, USA)	Pain VAS and the number of analgesics taken for 3 days post-extraction. Landry Wound Healing Index (LWHI) and H2O2 epithelization test. Intraoral periapical radiographs were obtained and analyzed using fractal dimension analysis to assess bone healing.	VAS scores at 1, 2 and 3 days: Control: 5.11 ± 1.6, 1.01 ± 1.44, 0 L-PRF: 3.3 ± 2.07, 0.48 ± 0.92, 0 T-PRF: 3.29 ± 1.85, 0.47 ± 0.62, 0	none	None
Bhujbal et al., [38], India	Cohort Study (III) - Moderate	Sites: 40 Males: 7 Females: 13 Age: 26 ± 6.9	Mandibular Third Molars	Sites: 20 Protocol: PRF Sites: 20 Protocol: PRF	PRP: 1200 rpm x 10 min with citrate phosphate dextrose adenine + 2000rpm x 10 min with calcium chloride PRF: 3000 rpm x 10 min	Landry’s healing index. Pain VAS. Swelling. Digital panoramic radiographs.	VAS scores at 1, 3 and 7 days: PRF: 1.8 ± 0.7, 2.2 ± 0.7, 0 PRP: 2.3 ± 0.8. 2.7 ± 1, 0.1 ± 0.2	none	None
Caymaz & Uyanik [37], Turkey	RCT (II) - High	Sites: 54 Males: 12 Females: 15 Age: (18–26)	Mandibular Third Molars	Sites: 27 Protocol: A-PRF Sites: 27 Protocol: L-PRF	A-PRF: 1500 rpm x 14 min (Elektro-mag M415P, Istanbul, Turkey) L-PRF: 3000 rpm x 10 min (Elektro-mag M415P, Istanbul, Turkey)	Pain VAS, and the number of analgesic tablets taken. Trismus and swelling.	VAS scores at 1, 2, 3 and 7 days: A-PRF: 31.56 ± 5.04, 15.67 ± 3.42, 7.03 ± 1.68, 1.67 ± 0.9 L-PRF: 48.3 ± 5.48, 32.44 ± 4.98, 19.01 ± 3.48, 3.22 ± 1.15	A-PRF	None
Unakalkar et al., [37], India	Cohort Study (III) - Low	Sites: 25 Males: NR Females: NR Age: (18–40)	Mandibular Third Molars	Sites: NR Protocol: PRF Sites: NR Protocol: PRP	NR	Pain VAS, trismus, and swelling were Periodontal health. Bone healing based on lamina dura score, density score, and trabecular pattern score. Duration of surgery, volume of local anaesthetic solution, and any intraoperative complications.	VAS scores at 1, 3 and 7 days: PRF: 6.33 ± 1.11, 4.07 ± 1.71, 1.73 ± 1.28 PRP: 5.93 ± 1.28, 3.73 ± 1.44, 1.47 ± 1.5	none	None
Dutta et al., [35], India	RCT (II) - Moderate	Sites: 40 Males: 27 Females: 13 Age: 27 ± 5 (17–36)	Mandibular Third Molars	Sites: 10 Protocol: Control Sites: 10 Protocol: HA Sites: 10 rotocol: PRF Sites: 10 Protocol: PRP	PRP:2000rpm x 15 min with citrate phosphate dextrose adenine + 3000 rpm x 10 min with calcium chloride PRF: 3000 rpm x 10 min with Calcium gluconate	Swelling, pain Wong-Baker VAS, dry socket Blum’s criteria, and soft tissue healing. Bone healing intraoral periapical radiographs.	VAS scores at 1 and 3 days: Control: 5.4 ± 1.7, 3.6 ± 1.13 PRF: 2.4 ± 0.75, 0.8 ± 0.25 PRP: 2.9 ± 0.91, 1.3 ± 0.41 HA: 5 ± 1.58, 3.6, 1.13	none	None

A table showing a summary of the characteristics and reported variables for each included study in this review.

*NR* Not reported.

*VAS* Visual Analogue Scale.

*rpm* Revolutions Per Minute.

*RCT* Randomised Controlled Trial.

*PRP* Platelet-Rich Plasma.

*PRF* Platelet-Rich Fibrin.

*L-PRF* Leukocyte-PRF.

*A-PRF* Advanced-PRF.

*T-PRF* Titanium-prepared-PRF.

*HA* Hydroxyapatite.

### Risk of bias

**Fig. 3:**
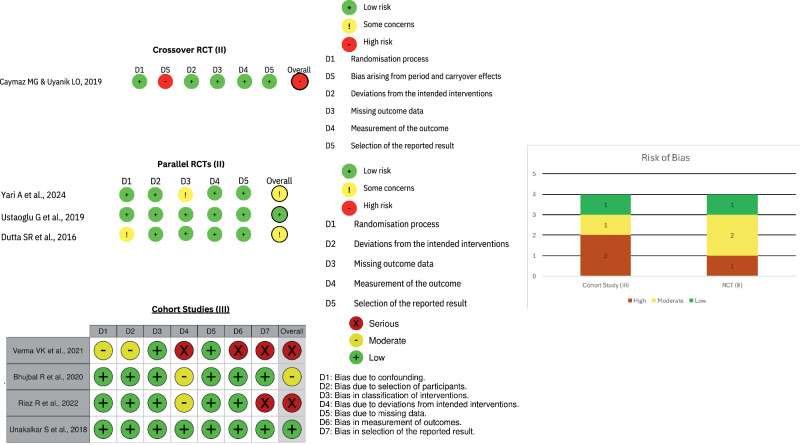
Risk of Bias Tools for the Included Studies.

The risk of bias assessment focused exclusively on patient-reported pain, excluding bias considerations related to other outcomes such as trismus, swelling or bone levels. Two studies exhibited a low risk of bias, three studies had a moderate risk of bias, and three studies showed a high risk of bias (Fig. 3). Despite the varying levels of bias, there was no evidence suggesting that any study was biased in favour of a particular protocol regarding postoperative pain scores.

### Pain

Owing to the small number of eligible studies (*n* = 8), the heterogeneity in pain measurement methodologies and the subjective nature of patient-reported pain; subgroup, sensitivity, meta-, or meta-regression analyses were not undertaken. Consequently, results of the studies were synthesised narratively, without statistical pooling, with a qualitative comparative network analysis.

**Fig. 4:**
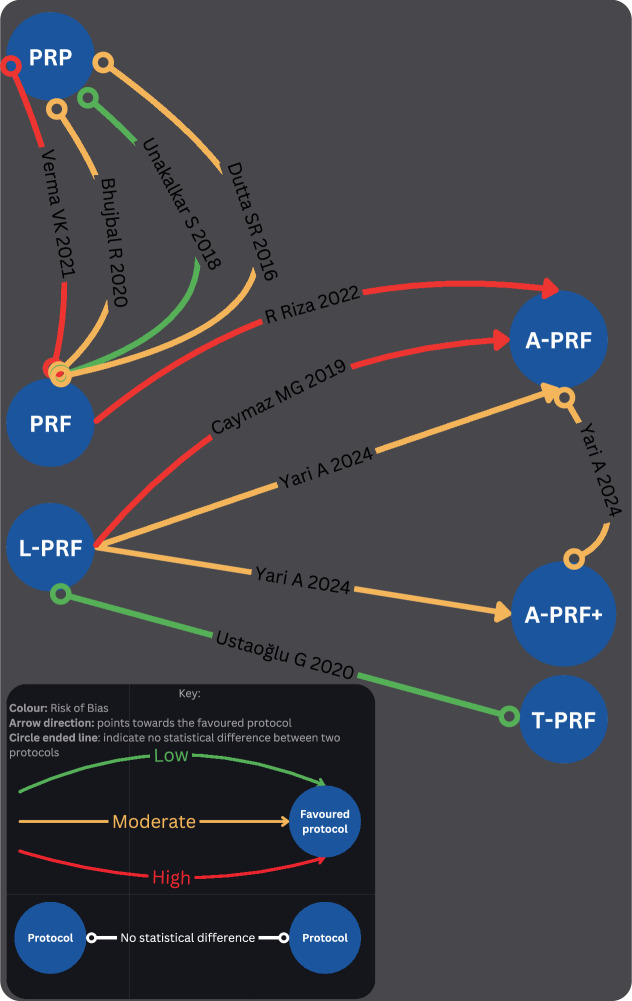
A Qualitative Comparative Network Analysis. A Summary of the comparative results in postoperative pain reduction between different APCs.

Four studies, with varying levels of bias (low, moderate, and high), have demonstrated no statistically significant difference in postoperative pain between platelet-rich plasma (PRP) and platelet-rich fibrin (PRF). One study indicated that advanced platelet-rich fibrin (A-PRF) is superior to PRF in reducing postoperative pain, with high risk of bias. Additionally, two studies reported that A-PRF is more effective than leukocyte-rich PRF (L-PRF) in alleviating postoperative pain on day 2, with moderate to high risk of bias. Another study found A-PRF+ to be more effective than L-PRF in managing postoperative pain with a moderate risk of bias, although no statistically significant difference was observed between A-PRF and A-PRF + . One study with a low risk of bias found no statistically significant difference in postoperative pain between leukocyte-rich platelet-rich fibrin (L-PRF) and titanium-prepared platelet-rich fibrin (T-PRF). (Fig. 4).

### GRADE

Table 3 reflects the summary of the GRADE assessment carried in this review. 6 out of 8 studies were assessed as having a moderate to high risk of bias. Nevertheless, there is no evidence to suggest that biases either reduce or increase the effect size between APCs. Additionally, no study presented results that contradicted those of another study and no study demonstrated a large effect size. Statistical test for heterogeneity could not be observed as results were quantitatively incomparable.

**Table 3:** Summary of evidence quality for APC studies on pain reduction.

	Studies	Risk of Bias	Inconsistency	Indirectness	Imprecision	Publication bias	Is there large effect size?	Is there dose response relationship?	Does confounding biases reduce the effect size?	Overall Quality
**Comments**	4 RCTs 4 Cohort studies	75% (6/8) moderate to high risk of bias	Since results were quantitatively incomparable. Point estimates, confidence intervals and statistical test for heterogeneity could not be observed. No study showed contradicting results to another study.	88% (7/8) of the studies focus on third molar extractions only. There is also a lack of head-to-head comparison between PRF and L-PRF	87% (13/15) of the individual results which showed statistically significant difference in pain scores at day 2 and 3 showed low to moderate imprecision/ variability. Since results were quantitatively incomparable, hedges’ d for size effect was incalculable and hence confidence interval for the effect size was not observed	88% (7/8) of the studies stated the authors did not have any relevant financial relationship(s) with a commercial interests. 12% (1/8) studies did not comment on the presence of external sources of funding.	No study showed a large effect size	Not applicable in the context of APCs	No evidence to suggest biases reduce or increase the effect size in favour for a particular APC	Low
**Score**	4	Serious (-1)	Not serious (0)	Serious (-1)	Not serious (0)	Not serious (0)	0	0	0	2

The table includes a breakdown of the GRADE criteria for assessing evidence quality.

Furthermore, 7 out of 8 of the studies focused exclusively on third molar extractions, highlighting a narrow scope in the research. A significant gap in the literature is the lack of direct comparison studies between L-PRF and PRF on postoperative pain reduction following tooth extraction.

**Fig. 5:**
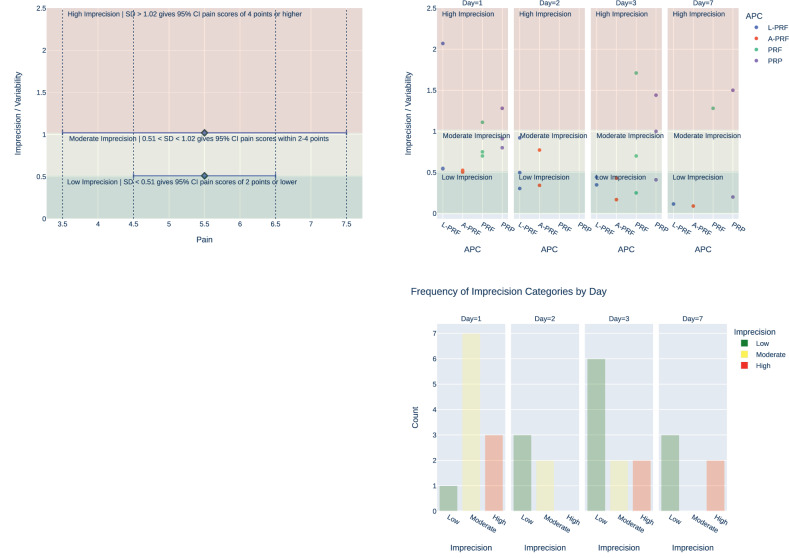
Analysis of imprecision in pain score measurements across different days and APC Interventions. The top left plot categorises pain scores into three imprecision categories—low, moderate, and high—based on the standard deviation (SD) and confidence intervals (CI). The top right plot shows the distribution of imprecision/variability across different days (Day 1, Day 2, Day 3, Day 7) for each intervention. The bottom plot displays the frequency of these imprecision categories by day.

Owing to the large heterogeneity described earlier—particularly differences in APC preparation protocols, subjective pain scales, follow-up periods and third-molar–only study populations—pooling data was neither statistically nor clinically appropriate. Instead, we qualitatively mapped the network of available comparisons and judged consistency by narrative synthesis. While this approach preserves clinical nuance, it inevitably yields wider uncertainty around the true magnitude of differences between any APCs. Consequently, the variability of individual results was assessed to categorise imprecision as low, moderate, or high. This categorisation was based on thresholds corresponding to how wide were the estimates of pain Visual Analogue Scale (VAS) results. Specifically, variability corresponding to a 95% confidence interval of 4 points or higher was considered high, variability within 2–4 points was deemed moderate, and variability of 2 points or lower was categorised as low. 13 out of 15 (87%) of the individual results, which demonstrated a statistically significant difference in pain scores on days 2 and 3, exhibited low to moderate imprecision/variability (Fig. 5).

There were insufficient comparative pairs of APCs data to construct a meaningful funnel plot or to conduct Egger’s test to assess for publication bias. Nevertheless, 88% (7/8) of the studies reported that the authors did not have any relevant financial relationships with commercial interests. In contrast, 12% (1/8) of the studies did not provide information regarding the presence of external sources of funding. Based on the evidence from the studies included in this review, the overall GRADE assessment of the quality of evidence has been determined to be ‘Low’.

## Discussion

The current literature on APCs supports their added benefits in socket preservation [17, 18]. However, in maxillary sinus augmentations, studies have demonstrated no additional benefits of APCs when combined with bone grafts [43–45]. Furthermore, Alrayyes (2022) highlights the lack of standardised preparation protocols for specific APC formulations [19].

Patient-reported outcome measures (PROMs), such as self-reported pain, are valuable tools for monitoring treatment responses and patient quality of life, particularly when collected soon after treatment. These measures aid in investigating clinical factors that enhance patient communication and satisfaction, ultimately improving patient engagement with the care plan provided [46–48].

Since pain is likely to be the first postoperative complication, starting from day 0 and lasting up to day 7 [20], it is valuable for patient satisfaction and quality of life to determine strategies to reduce it.

Research by Mourão (2020) shows that L-PRF improves healing and decreases pain following tooth extraction compared to controls [49]. A systematic review by Al-Maawi (2021) confirms the efficacy of PRF over control in reducing postoperative pain [50].

Our review explored comparative studies between different APCs regarding their efficacy in postoperative pain reduction, revealing that A-PRF and A-PRF+ offer additional benefits over L-PRF and PRF, with no significant difference between PRF and PRP.

### Implications for clinical practice

For tooth extractions, APCs can be applied to extraction sites to promote faster healing and reduce postoperative pain. Nonetheless, there are practical limitations for using APCs in dental practice:

1. The preparation of APCs requires specialised equipment, which can be costly.
2. Dental practitioners need specialised training to effectively use APCs. This includes understanding the preparation, handling, and application of APCs, since different centrifugation speeds, times, and techniques can produce varying qualities and quantities of APCs [8].
3. Patients with blood disorders such as thrombocytopenia may not be suitable candidates for APCs therapy since their platelets are compromised. Likewise, conditions such as diabetes, immunocompromising diseases and chronic infections can impact the body’s healing response [51, 52] and as a result could reduce the effectiveness of APCs treatments.
4. Medications such as anticoagulant and antiplatelet (e.g., warfarin, aspirin) and nonsteroidal anti-inflammatory drugs (NSAIDs) can affect platelet function [53] and hence could reduce the overall effectiveness of APCs. Furthermore, the use of corticosteroids may interfere with the body’s healing process [52] and thus may affect the efficacy of APCs.
5. Patients must be fully informed of the relative novel nature of APCs and the potential efficacy and benefits involved.

Evidence identified in this review supports A-PRF as the most effective APC for postoperative pain reduction following tooth extraction, however, with a ‘Low’ GRADE recommendation level. This means that the estimated effect of an intervention may be substantially different from the true effect. Consequently, further clinical research is necessary to support a strong recommendation for the general practice use of A-PRF.

### Limitations

The inclusion criteria limited the selection to studies available exclusively in English, potentially excluding articles that might either support or contradict the conclusions of this review. Additionally, the temporal range of considered articles, from January 2014 to June 2024, may not consider all relevant developments in the field. In addition, only eight studies met the eligibility criteria, thereby limiting the generalisability and statistical power of the findings. Also, seven out of eight studies focused exclusively on third molar extractions, which further narrows the clinical applicability of the results to other types of tooth extractions. Furthermore, the lack of direct comparisons between PRF and L-PRF highlights a gap in the literature that reduces the comprehensiveness of the review. Another important limitation is the inherent heterogeneity among the selected papers, possibly arising from variations in methodologies, populations, or APC preparations, introducing potential inconsistencies: centrifugation speeds ranged from 1300 rpm (single-spin PRF) to 3000 rpm (second spin in two-step PRP protocols) with total spin times of 8–15 min; several studies employed sequential double-spin methods. Preparations differed in both leukocyte content (e.g., L-PRF vs. PRF) and anticoagulant use (present in all PRP arms but absent from PRF-type concentrates). Pain was measured with either 0–10 or 0–100 visual-analogue scales at postoperative intervals spanning 1 to 15 days. Surgical difficulty and operator experience were seldom reported. These discrepancies limit direct comparability and likely underlie the dispersion of point estimates observed in Fig. 4. Moreover, a notable constraint is the presence of moderate-to-high risk of bias within 75% of the selected papers, coupled with a ‘Low’ GRADE score for evidence quality assessment, which could affect the overall validity and reliability of the synthesised findings. The inability to conduct a meta-analysis impeded a robust evaluation of imprecision using a combined confidence interval and hindered a robust assessment of publication bias through funnel plots or Egger’s Test. Finally, the present review was not previously registered, which increases the risk of an unplanned duplication.

### Recommendations for future trials

The current literature clearly demonstrates the benefits of autologous platelet concentrates (APCs) in pain management, socket preservation, bone regeneration, and promoting healing compared to controls. Future trials should focus on comparing different APCs to each other to identify the most effective APC for achieving specific benefits in particular clinical settings. Furthermore, it would be beneficial to investigate which patients could derive the most benefit from the procedure by identifying specific patient characteristics predictive of a favourable or unfavourable response. This knowledge would aid practitioners in case selection for implementing APCs.

In the context of tooth extraction, this review identified limited evidence suggesting that A-PRF and A-PRF+ are more effective in reducing postoperative pain compared to L-PRF and PRF directly, and PRP indirectly. Notably, none of the eight eligible trials offered a direct head-to-head comparison between the conventional PRF and its leukocyte-rich variant (L-PRF). This omission represents a gap in the literature.

## Conclusion

Between January 2014 and June 2024, 8 clinical studies have been identified that compare the efficacy of different APCs for postoperative pain management following tooth extraction. A-PRF and A-PRF+ were favoured to reduce postoperative pain on day 2 among the investigated APCs, although the GRADE criteria rate the evidence as “Low”. This conclusion must be interpreted cautiously in view of large heterogeneity in the studies’ methods and follow-up periods (1–15 days). Future trials should directly compare A-PRF or A-PRF+ with PRF and L-PRF using high-quality randomised-controlled designs.

## Supplementary information

PRISMA checklist

Supplement 1b

## Data Availability

The data that support the findings of this study are available from the corresponding author upon reasonable request.
