# Supplementary material for: Patient reported pain following tooth extraction with different autologous platelet concentrates. Systematic review
Source: BDJ Open. 2025 Jul 17;11:66. doi: 10.1038/s41405-025-00348-2 (PMC12267419; doi:10.1038/s41405-025-00348-2)
Supplement: Supplementary file 2 — Supplement 1b [file 41405_2025_348_MOESM2_ESM.docx]

**Supplement 1b**. Inclusion and exclusion criteria.

A table showing the inclusion and exclusion criteria used during the articles selection process which led to the final inclusion of the studies in this review.

| **Inclusion criteria** | **Exclusion criteria** |
| --- | --- |
| - Hospital or clinical setting - Adult population (18-75 years old) - Randomized controlled trials or Cohort studies - Studies utilising APCs in extraction sockets - Studies reporting patient reported pain - Studies comparing at least two different APCs - The investigation period is between 01^st^ of January 2014 and 24^th^ of June 2024 - No restrictions on type of APCs - No restriction on study sample size - No restrictions on geographic location | - Studies compare only one APC to control(s) - Studies not reporting patient reported pain - Paediatric or special needs population - Restorative or endodontic treatment - Non-dental procedures - Animal studies, books, reviews, case reports, editorials, letters, commentaries, or conference abstracts - Duplicate studies - Non-English articles |
